# Experimental and computational analysis of mouse sleep-wake dynamics

**DOI:** 10.1186/1471-2105-13-S12-A21

**Published:** 2012-07-31

**Authors:** Farid Yaghouby, Ting Zhang, Martin Striz, James Crawford, Kevin Donohue, Bruce O’Hara, Sridhar Sunderam

**Affiliations:** 1 Center for Biomedical Engineering, University of Kentucky, Lexington, KY 40506, USA; 2 Department of Biology, University of Kentucky, Lexington, KY 40506, USA; 3 Department of Electrical and Computer Engineering, University of Kentucky, Lexington, KY 40506, USA

## Background

Genetic and behavioral screening of mice play important roles in sleep research, but the need for invasive electrophysiological (EEG/EMG) measurements for determination of sleep-wake and behavioral state limits the scope and rate of experimentation.

## Materials and methods

In this study we explore the utility of a noninvasive method based on the signal from a piezoelectric sensor on the cage floor for scoring sleep-wake behavior in mice. It was previously demonstrated that the piezo signal can accurately discriminate sleep from wake activity; however, this was verified mostly by visual observation. Here we perform a more objective validation by correlating piezo measurements with EMG activity, which is dramatically suppressed during sleep. Furthermore, the piezo sensor is sensitive to respiration-related thoracic movements. Since breathing is relatively irregular in REM sleep compared to non-REM, we extract piezo features that reflect breathing regularity to try to distinguish between these sleep states.

## Results

We validate our methods against simultaneous video/EEG/EMG measurement, which constitute the gold standard for scoring sleep. But rather than rely on subjective visual scoring to determine state, we use an unsupervised probabilistic model, the hidden Markov model (HMM), to automatically partition time series of extracted EEG/EMG features into REM, non-REM and wake states. A similar HMM, estimated exclusively from piezo features of instantaneous energy and breathing regularity, displayed dynamical stages with a similarity to REM/non-REM sleep, transient arousal, and wakefulness. These preliminary results suggest that a combination of piezoelectric measurements and computational modeling could yield a novel noninvasive method for analysis of sleep and sleep-related disorders.

